# Supplementary material for: EEGs Disclose Significant Brain Activity Correlated with Synaptic Fickleness
Source: Biology (Basel). 2021 Jul 11;10(7):647. doi: 10.3390/biology10070647 (PMC8301300; doi:10.3390/biology10070647)
Supplement: Supplementary file 1 [file biology-10-00647-s001.zip › biology-1250399-supplementary.pdf]

Supplementary material

EEGs disclose significant brain activity correlated with synaptic fickleness

May 22, 2021

1 The role of E-E and I-I connections.

The original model was designedly kept minimalist, following [1, 2], in order to extract basic properties of a system in which excitatory and inhibitory counterparts interact to produce oscillatory phenomena of different nature, and thus internal E-E and I-I were initially ommited.

When local recurrent connections between excitatory neurons in the network are included, the observed effects can be divided in two; first, a larger number of afferent excitatory neurons translates into an increased overall excitability of the network, leading to the appearance of oscillatory behavior for lower levels of noisy input, as well as to a shift of the transition point of the explosive transition to lower values of μ (see Figure 1). Secondly, a larger connectivity between excitatory cells leads to increased spike-timing correlations, resulting in an enhanced amplitude of the oscillations (see figure 1right). Alternatively, the presence of I-I connections results in a decreased activity of the inhibitory population, reducing the amplitude of the oscillations and narrowing down the difference between the two branches of the hysteresis loop (see Figure 2). However, the phase transition and the hysteresis loop remain if the I-I connectivity is not very large.

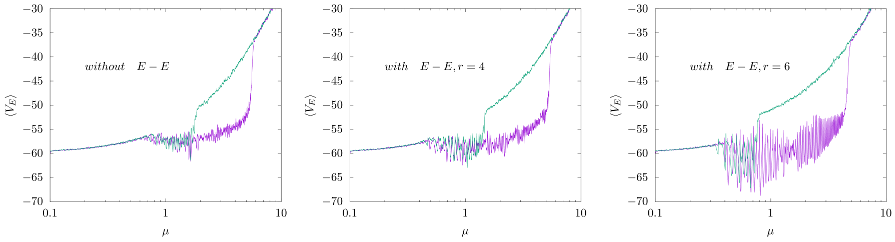

Figure 1: Influence of E-E connections on explosive transition and hysteresis emergence. The presence of E-E connections makes the brain waves to emerge at lower values of the external noise μ and to increase their coherence. Following the original regular network topology in [1, 2], we consider each excitatory neuron is influenced by other excitatory neurons located in the network within a circle around it with radius r, being $r = 1$ the semidistance between neighbor excitatory neurons. The panels corresponds then, from left to right, to increasing number of excitatory afferents corresponding to $r = 0, 4, 6$,respectively.

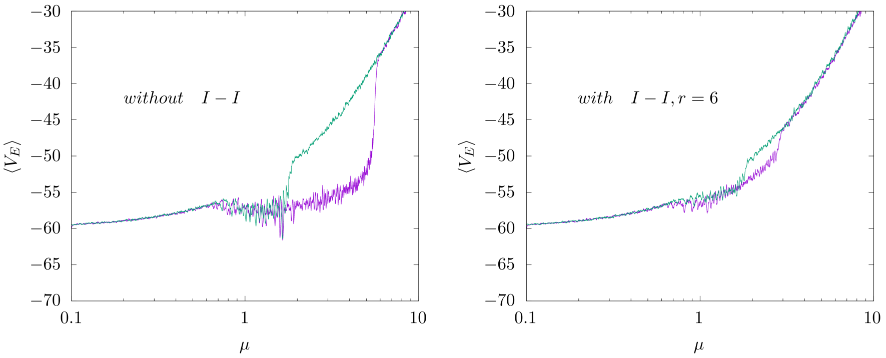

Figure 2: Influence of I-I connections on explosive transition and hysteresis emergence. In this case, the presence of I-I connections makes the brain waves to emerge at larger values of the external noise μ and to diminish their coherence. However, the explosive transition and hysteresis still is present when one consider a larger number of inhibitory afferents of the inhibitory neurons (i.e. $r = 6$), although in this case the transition is less evident and hysteresis loop is narrower. As in case of E-E connections above, we consider here that each inhibitory neuron is influenced by other inhibitory neurons located in the network within a circle around it with radius r.

2 The role of network size on the emergence of the explosive transition and hysteresis loop.

In order to test the robustness of our main results when the network size is enlarged, the adiabatic variation of the noise level μ (see Results Section in the main text) was repeated for systems constituted by $N = 180$, 320, 500, 2000 neurons, respectively. As depicted in Figure 3, one clearly observe that the phase transition and the hysteresis loop remain nearly unchanged when the network size is increased, so it is therefore likely to occur even for very large neuron populations.

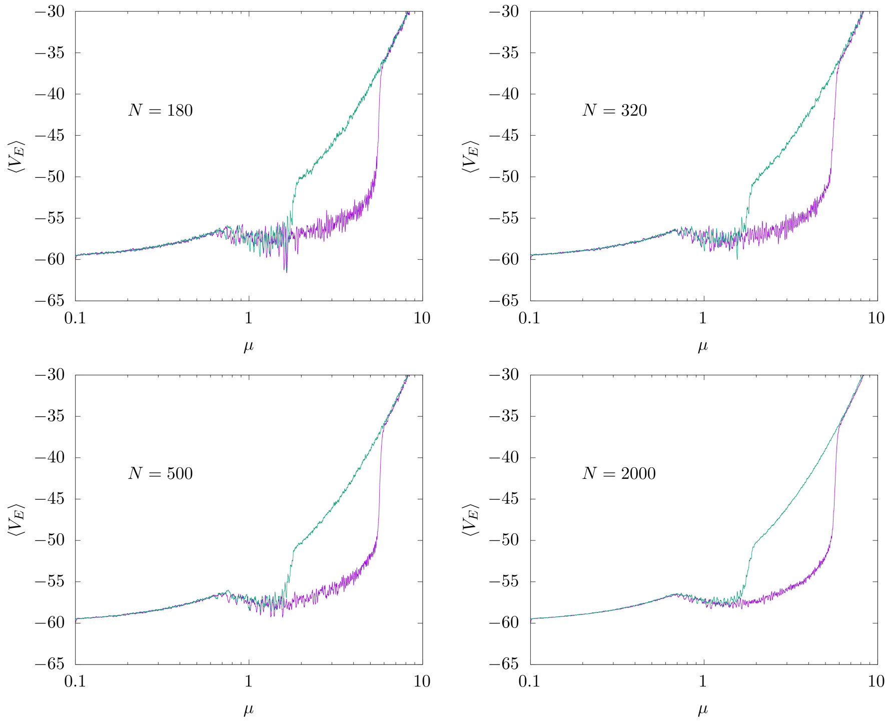

Figure 3: Robustness of the appearance of the explosive first order transition and hysteresis loop when the size of the neuron populations is increased. The figure illustrates that the relevant phenomenology in our system does not depends on the network size.

Also the features of the oscillatory behaviour just around the explosive transition is not dramatically affected by the network size as it is depicted in figure 4

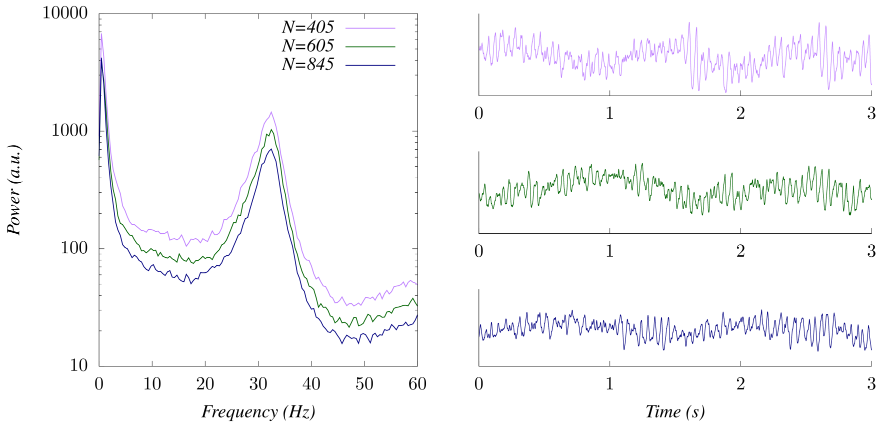

Figure 4: The effect of increasing network size on the features of the emerging waves near the explosive transition ($\tau_{rec} = 230ms$). This analysis illustrate that the low frequency modulation of high frequency waves are robust and do not change when network size is increased as different power spectra show (left). Time series of the emerging waves from which the power spectra have been computed (right) clearly depicting this low frequency modulation for all network sized considered.

3 The role of the network topology

Lastly, to observe the effect of topological irregularities on the emergent phenomena, the existing E-I and I-E connections from excitatory to inhibitory neurons in the original network were randomly rewired to any E and I neurons in the system with probability p_r, leading to a small-world-type connectivity pattern. The results of this analysis are summarized in figure 5. This illustrates that both the existence of the explosive transition in the presence of dynamic synapses and the emergence of hysteresis is not dramatically affected by the rewiring of neuron connections.

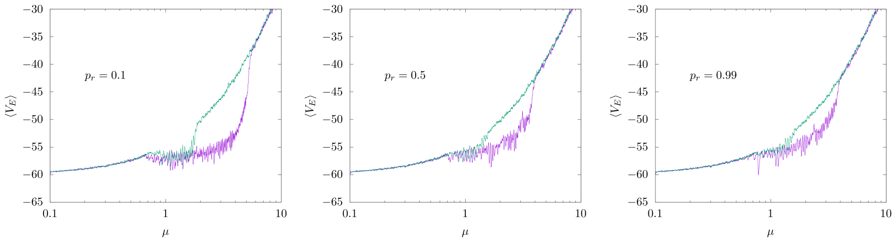

Figure 5: The role of topology on emergent phenomena in our system. We rewire the original regular topology in our system with some rewiring probability p_r to visualize the robustness of the explosive transition and the appearance of hysteresis in the oscillatory behavior of our neural network with dynamic syanpses. Our study illustrate that even in the case of random E-I and I-E connections our system displays the emergence of brain waves and an explosive transition when μ is increased with the presence of hysteresis.

References

[1] F.H. Lopes da Silva *et al.*, “Model of brain rhythmic activity; the alpha-rhythm of the thalamus”, *Kybernetik* 15, 27 (1974)

[2] J.A. Galadí *et al.*, “Emergence and interpretation of oscillatory behavior similar to brain waves and rhythms”, *Communications in Nonlinear Science and Numerical Simulation* 83, 105093 (2020)
